# Experiences in the Treatment of Gonorrhœa*Read before the Bœnninghausen Club

**Published:** 1895-04

**Authors:** Olin M. Drake

**Affiliations:** Boston, Mass.


### EXPERIENCES IN THE TREATMENT OF GONORRHŒA.*

OLIN M. DRAKE, M. D., BOSTON, MASS.

MR. CHAIRMAN, COLLEAGUES, AND FRIENDS:—I have now been treating gonorrhœa for twenty-five years. In my former field of practice, in Maine, my experience with this disease was quite extensive, considering the population of the city and neighborhood. It was not unusual for me to have on hand, at one time, from eight to ten cases. After a *fête* day or the passage through our city of a circus, my coffer would be replenished from the revenue of a host of clap patients. Since I have taken up my residence under the classic shades of the State House, my practice in the venereal line has not been so large, but the cases I have had have been so interesting that I shall mention some of them before concluding this paper.

Gentlemen, no one among you more fully realizes than I do, that we have gonorrhœa and gonorrhœa to treat—in other words, that we have cases of simple urethritis and cases with a sycotic basis. The former are easily cured by practitioners of both schools; but as regards the latter, the cure of many of them is often a task of great patience and skill, and only obtained by our school, unless the allopathic practitioner accidentally hits upon the remedy, homœopathic to the case.

I have been successful in all cases of gonorrhœa where the patient followed my directions and pursued the treatment I ordered. In fact, my success has been so marked that I have not seldom asked myself whether I had really ever attended gonorrhœa with a sycotic basis. My knowledge of disease convinces me that I have, but of course I may be mistaken. When such truly able Hahnemannians as Lippe, P. P. Wells, William A. Hawley, Biegler, and William P. Wesselhœft have admitted that they have often experienced the utmost difficulty in curing some of their cases of gonorrhœa, I must confess I feel some

---

* Read before the Bœnninghausen Club.

hesitancy in relating my experiences; but these records are honest, gentlemen, and reflect the truth, the whole truth, and nothing but the truth.

I have had very many cases of gonorrhœa that have come to me, after months and even years of allopathic drugging; cases, too, with prostatitis, orchitis, strictures, gonorrhœal rheumatism, etc., and I honestly believe I have cured them all, with the exception of a few, of which more anon. Of course, some of my patients may have deceived me and gone elsewhere for treatment; if they have, it is not to my knowledge; but in most cases I know for a fact that they were positively and absolutely cured.

According to Lippe, whose remarkable ability as a clinical observer has not been surpassed in our school since Bœnninghausen, suppressed gonorrhœa cannot be cured, and he makes the confession "he has never cured such a case yet"—(*vide* HOMŒOPATHIC PHYSICIAN, Vol. VII, p. 283). We may think, he continues, that we have succeeded in doing so, and for years this will seem so to the patient and physician, but suddenly, when we least expect it, the disease, "like the locust in seventeen years," inevitably returns.

With all due respect to our late lamented teacher, I believe he was mistaken, and I base this conclusion upon twenty-five years of experience. I should like much to hear the views of some of the members of the club on this point. Of course I can imagine the sycotic, engrafted upon some psoric or syphilitic constitutions, proving incurable, but such cases must be the exception.

In giving you the particulars of my mode of treatment in gonorrhœa, I am aware that I shall lay myself open to adverse criticism; for my rule, I believe, varies from what most of you follow. However, the aim of us all is to honestly state our methods and experiences, with the view of comparison and ultimate benefit, and I frankly state my practice.

If the patient presents himself after the discharge has already existed for a day or two, with slight redness of the meatus and dysuria, *without any characteristic symptoms indicating a par-*

*ticular medicine*, I invariably commence the treatment with *Cannabis-sativa*$^{200}$ (Dunham's). I order four powders a day for four days; after this I give *blanks* for ten or more days, unless decided symptoms, calling for another medicine, appear. In this way and with this medicine, and with such repeated doses, I have cured very many cases. If, after the expiration of ten days or thereabouts, I see no further improvement, I proceed to the re-examination of my patient, and I spare no pains in making it most thorough. I note down in writing, his history, that of his ancestors, and sometimes that of his "sisters, his cousins, and his aunts," with all his symptoms, conditions, etc.

I take plenty of time before prescribing a second time, and not seldom I send my patient off with blanks, until I am certain I have made the best selection within my poor ability. This second remedy I give as I did the first—in repeated doses—to be followed by blanks. If, however, my researches satisfy me the first prescription is still the best, I give a single dose of it, of a higher potency, and await results.

In the earlier years of my practice—some twenty years ago—I generally resorted from the 3d$^{x}$ to the 6th$^{x}$ attenuation, if I had to repeat my medicine, but I am convinced now that it would have been better had I given higher potencies still, as I do at present.

The late Dr. Henry N. Guernsey, whose friendship I enjoyed and to whom I am indebted for many kindnesses, as well as valuable therapeutical hints, told me that he gave only one dose of the indicated remedy in gonorrhœa, and of a high potency. He assured me that the results were all that he could desire. By the bye, he has left us an excellent article on gonorrhœa, which I cannot too strongly urge those who are not familiar with it to read (*Hahnemannian Monthly*, Vol. XIII, p. 343). I have often tried the single dose plan, but somehow or other I have been disappointed in it, and yet this much esteemed pioneer of Homœopathy was an honest and reliable teacher.

As a rule it rarely takes me more than seven weeks to cure gonorrhœa, but I have cured many cases in considerably less time. I have dismissed, *cured*, the worst case of gonorrhœa I ever met, with profuse discharge, phymosis, chordee, and general inflammatory symptoms, after two prescriptions; but I remember one case, the subject being a perverse old sinner, when it took me about two years to cure him; but, in justice to myself, I must add, that this arrant reprobate "reclapped" himself five or six times during that period.

*Apropos* of this fallible mortal, who loved the sex too well but not wisely, he unfortunately gave his wife, some years ago, the clap while she was pregnant. Sometime after her confinement, during which she was attended by an old-school doctor. I was requested, by her physician, to take charge of her baby, who was suffering from gonorrhœal ophthalmia. The trouble had been going on for eleven days and the doctor felt sure his little patient would lose her eyesight. I found both eyes greatly swollen, nearly the size of a pullet's egg; conjunctiva of both eyes and eyelids intensely red, the chemosis so marked that the corneæ seemed to recede fully a quarter of an inch. On attempting to separate the eyelids, a profuse flow of yellow pus gushed out, and the *whole* cornea, of both sides, was *pearl-colored*. I felt, myself, that the sight was hopelessly gone, and so informed the parents. I gave Argentum-nitric$^{200}$ (D.) internally, and the same potency in water to be used locally. I would not now use the remedy locally. Extraordinary as it may seem, the recovery was complete. A year ago last summer I saw this same patient, who had, in the interval, blossomed into a beauty, fifteen years old, and I give you my word, gentlemen, I could not detect the faintest trace of a spot or speck upon either cornea.

While on the subject of lewd sinners, let me relate another instance. A farmer, of hitherto exemplary domestic fidelity, was tempted by the wiles and fascinations of a buxom servant girl, and the poor devil, unlike St. Anthony of old, succumbed. Presently, however, he felt the necessity of consulting me, and I diagnosed a beautiful case of clap. But what was worse still, he feared he had communicated the disease to his wife, and, unfortunately, this proved to be true. Now this spouse was decidedly muscular, had the temper of a virago, and withal she could handle a broomstick with a good deal of vigor, as well as skill. She had also very penurious habits. This had been, in the past, a gratification to her husband, but to-day it interfered with his plans. She did not believe in consulting a doctor for such a trifle as dysuria and leucorrhœa, and say what he could she would not come to me. As she was strong and healthy otherwise, her natural instincts would manifest themselves from time to time, and then the well-trained husband had to comply with her wishes.

He would plead fatigue, sleepiness, lack of inclination, but it was all of no avail, he had to perform his marital duties. To tell his wife the truth, was out of the question, for, he said, such a disclosure would mean nothing less than six months in the hospital. If ever a man did rack his brains to give his physician a job, this man did.

For six months I had this poor wretch under my care; he had no sooner began to improve when, to all appearances, he would have a fresh attack of gonorrhœa. I had a bonanza, meanwhile; I think he must have paid me nearly $100 during that time. I can see him now, walking up and down in my office, pulling his hair, swearing at the obstinacy of his wife, and vowing the poorhouse was staring him in the face. Finally his wife had a very severe attack of neuralgia, and I had my opportunity to rescue an unfortunate from the slough of despond and misery. I took the totality of the symptoms, to be sure, when prescribing for her, but I must admit I laid uncommon stress upon the urinary symptoms. I remember her impatience when I inquired into the latter symptoms, for she could hardly see any connection between the two; but soon afterward peace and happiness reigned once more by that fireside—way down in Maine.

As regards complications arising in the course of treatment, when I had the case from the beginning, I rarely had any, and when I had, they were easily controlled. I will relate, further on, the only case of this kind, where orchitis, in a mild form, was a complication.

I have no experience to give concerning the treatment of gonorrhœa in the initiatory stage by the so-called specifics. It may be well, however, to remember that Jahr advises Sepia[30]; Granvogl, Natrum-sulph.; Whale, Bryonia; Baehr, Mercurius-sol.; Kafka, Sulphur, etc. As for dietetic rules, I order the patient to observe a light diet—no meat or coffee, no spiced food or stimulants in any form, and I tell him to walk or exert himself as little as possible. I have often seen severe aggravations of the symptoms from a glass of beer, also from coition or the company of lewd females, as sexual excitement under such circumstances is generally prejudicial. I have, however, cured many subjects, who, in spite of my commands, used liquors, cohabited, smoked, and indulged in late suppers and dancing.

Such, gentlemen, is, in brief, the course of treatment I pursue in gonorrhœa. For years I studied my cases with the view of ascertaining if there were any symptoms upon which we should lay particular stress in the search for the simillimum; but I have long since concluded that in this, as in all diseases, we must treat every case upon its own individual merits. The symptoms most important are those which individualize that case from all others.

I well remember having a patient, some twelve or fifteen years ago, who had run the gauntlet of doctors and "cure-alls" for a gleet which he had been troubled with for nearly four years. He began by inquiring how long it would take to cure him. I gave him the answer I give every patient that asks the question: "It is impossible for me to tell you anything about it—it may take me six weeks and it may take me years. I will do the best I can by you, and let us hope for a speedy cure." Every week or two, under this compact, he would call at my office and I would prescribe as carefully as was in my power. I attended him, month in and month out, without any change in his condition, until we were both perfectly disgusted, and still that everlasting drop in the morning remained. He had no pain, only a too-frequent urination, in small quantities, the discharge was a glairy mucus, occasionally taking a greenish tinge, and it would agglutinate the meatus. One day while I

was questioning him particularly about the frequency of urination, he said that there was one thing "funny about it;" he could never hear running water without feeling a desire to pass his own water. In driving to town that morning, he added, he let his horse drink twice by the wayside, and when he heard the water pass down the horse's gullet, he had to get down from his cart and urinate; and this he had noticed previously. I immediately exclaimed "Eureka!" I had discovered the characteristic symptoms at last. One prescription of Lyssin$^{200}$ removed the whole trouble. And thus I have often found that the *key-note* to the case was, not unfrequently, some insignificant, absurd symptom, so trivial in the opinion of the patient that he would not mention it, unless by accident it was drawn out of him. Dr. Berridge recently reported a case in point. A patient troubled with an old-standing gonorrhœa, which had shown itself rebellious to his treatment, mentioned that he would have erections while riding in a car if he did not engage in conversation. He was cured by Calcarea-phos., the above symptom being the key-note.

A member of this club asked me, the other day, if any of my gonorrhœa patients "had given me the slip." I am aware of but few who have, as I have stated earlier in this paper. The first, who was suffering from gleet, did not return to me after his first visit, saying that he did not intend losing valuable time taking "white sugar." I know, for a fact, that fourteen months later that man was still consulting a neighboring allopathic physician for the same complaint. A second one left me after some months of unsuccessful treatment for chronic gonorrhœa. I cannot help admitting to you, gentlemen, that my failure on this occasion was due to my ignorance, and I make the confession with some qualms of conscience. I hope and believe I could do better to-day; but I did the best I knew at the time.

Unfortunately for humanity, there are, among our adherents, men who do worse, who injure their patients with injections, but I am happy to say I cannot charge my conscience with that sin. It is a matter of great regret that, even to-day, there are erring physicians, who, in the words of Hahnemann himself,

"would wish to be considered homœopathicians, and graft some, *to them*, more convenient allopathic bad practices upon their nominally homœopathic treatment," in consequence, as in his day, "of ignorance of the doctrine (homœopathic), laziness, contempt for suffering humanity * * * and of unpardonable negligence in searching for the best homœopathic specific for each case of disease," etc.

With most people afflicted with gonorrhœa, the thought uppermost in their minds is, how can they wipe out that "damned spot," which, like Banquo's ghost, is ever an object of dread, and often "will not down." They little realize the manifold train of serious symptoms which may follow the suppression of this discharge. I well remember the incredulous smile with which I heard the late Dr. H. N. Guernsey insist upon this undoubted fact, so ably brought to light by the noble founder of our school; but I have lived long enough, I am thankful to say, to realize how foolish I was.

Since my advent among you in the metropolis of New England, I was called one day to attend a young man, whose painful sufferings had lasted nearly three weeks. The medical attendant had diagnosed the patient's ailment, acute articular rheumatism. In spite of all his ministrations the subject continued to grow worse. Finally the patient's father insisted upon my being called in. The trouble had begun in the right knee, then it affected the right ankle, and soon both elbows, right wrist, and hand were involved; the joints were dark red, hot, and swollen; the pains sharp and decidedly aggravated by motion or the jarring of the bed; the exacerbations were generally in the forepart of the night; there was great and almost continued thirst; the urine high colored and scanty, etc. In spite of this picture hardly recalling a typical attack of gonorrhœal rheumatism, since so many joints were simultaneously affected, and the local inflammatory symptoms were so marked, yet I had an instinctive feeling that gonorrhœa was at the bottom of the trouble. I soon elicited the fact that he had but recently recovered from an attack of gleet which had lasted him for over two years. When I informed him that he must not look for any relief before the discharge returned, he expressed incredibility and disgust. To begin with, he was sure he had been cured five weeks previously by a New York physician (a specialist), by means of injections; and secondly, he added, in rather piteous tones, he could not afford to have the old trouble back, for it had already cost him $400. I prescribed Bryonia$^{200}$ (D.) in water. In less than thirty-six hours his penis was running as freely as a young maple tree in early spring. I never saw a more profuse discharge. In a few hours he would saturate a large place on a towel, and this lasted nearly ten days. From the moment the discharge began, the rheumatic pains diminished and the general condition improved. Two days later—although the swelling was much less—there was a good deal of soreness and sensitiveness to touch of the parts, and any jar aggravated; the discharge was still profuse, thick and of a yellowish-green hue; smarting of the urethra during urination was now present and there was much heat of the body in the evening as well as some thirst, but the constant thirst had discontinued. I now gave Pulsatilla$^{200}$ (D.) Steady but slow improvement followed, and at the expiration of nine days the discharge was found less and thinner and of a greenish instead of a yellowish-green tinge, and the pains in the articulations were numb in character, instead of acute. After a most careful study of the symptoms, comparing minutely Pulsatilla and Thuja, I decided upon the latter, and gave it in the 200 (D.) potency. Nine days later, although patient and his friends were satisfied with the progress in the case, I was not, and I repeated the Thuja in a higher potency. I waited five days more after this, when, seeing no further gain, I again gave Pulsatilla. Seven days now expired, during which his condition varied, but on the whole the patient seemed on the mend. He now began to be troubled with pains in his testicles, and this during two successive nights, when I administered Hamamelis$^{200}$T. This medicine seemed to relieve him in a certain degree, but eight days later, after another study of his case, I determined upon giving Pulsatilla again. Soon marked improvement set in, and in a fortnight afterward the patient seemed almost well, when I had to wind up the case with one dose of Medorrhinum$^{cm}$ (F.) dry, on the tongue.

I know that most of you have noticed the mistake I made on the eighth day of treatment of the above case. If anybody else had changed the medicine under the circumstances, substituting Thuja for Pulsatilla, when actually the patient was steadily—if slowly—improving I would have said, "How foolish," and yet that is precisely what I did.

It is the old story, my dear colleagues; criticism is an easy matter, but art is difficult. When a physician is very anxious to hasten the cure of a patient, he is likely to do the opposite. I should either have repeated the Pulsatilla, or, better still, allowed it to act undisturbed. Of course, I endeavored to repair the mischief later on, but fourteen days had elapsed meanwhile. By exactly that period of time this man's illness might have been shortened. At any rate, that is what I am inclined to think.

Something over a year subsequently this same patient contracted gonorrhœa again. Oh! for the frailty of human nature! The discharge, when he consulted me, which was seventy-two hours after its appearance, was slight and light in color, with heat of urethra during micturition, and inflamed meatus. I gave him Cann-sat.$^{200}$ (D.), in my usual way, with blanks to take afterward. On the twelfth day following the discharge was thick, yellowish-green, with frequent desire to urinate; burning urethra during micturition; occasional fine-stitching pains in prostatic portion of urethra; and right testicle swollen and sensitive, with dragging pains in the spermatic cord of the same side. I prescribed Pusatilla$^{200}$, of which I gave him sixteen powders, and a good supply of blanks, as I intended leaving the city the next day on my summer holidays. I ascertained on my return to Boston that he began to improve immediately after beginning the Puls., and was as well as ever in a short while, and this without any other medicine. By the bye, this is the only case I ever had where complications arose in the course of treatment where I had the patient under my care from the very outset.

The next case of gonorrhœa I shall relate is one of exceeding interest, and admirably exhibits the marvelous results of homœopathic medication. The subject had an attack of gonorrhœa nine years previously, during which time he received heroic allopathic treatment. It left him with a stricture of the urethra, which, notwithstanding forcible dilatation, and even incision, impeded the expulsion of urine, rendering its passage difficult and the stream very small. There was present, immediately over the stricture, situated about one and a half inches from the meatus, a thickening or induration the size of a bean, and from this stricture an almost continual discharge of mucus. At intervals, for severals days at a time, he would become despondent, during which attacks he would cry like a child, seek solitude and long for death, even planning means by which he could accomplish his purpose. In spite of all these troubles, some fair inamorata, early in June, 1893, inveigled him, by her sweet smiles, from the path of virtue, and your humble servant had a few days later another patient on his hands.

On the fourth day of the above month the patient presented himself at my office, with the following symptoms: Frequent desire to urinate, night and day, with much burning in urethra, especially forepart and orifice, during urination, the burning lasting for a short time afterward; lips of meatus and surrounding tissue of glans inflamed; discharge clear and glairy, but not profuse. My prescription was Cann-sat.$^{200}$, sixteen powders and blanks. His condition was pretty much the same until the 20th, when he complained of much irritation in posterior part of urethra, extending into rectum, with almost constant desire to defecate; he had also painful erections at night, with profuse thick, yellow discharge. I gave him Nux-vom.$^{200}$. On the 25th he reported that he had been better, but was not quite so well since the day before. I repeated Nux-vom. in the 50 M. During the middle of the following night I was roused to answer a call from this patient. He appeared greatly disturbed, mentally and physically, and behaved like one bereft of his senses. He wept like a child and declared he would go and drown himself if I did not give him immediately some Morphine; meanwhile he was walking up and down my room, with a very wild expression in his eyes. But I was not just then in a mood to submit to any nonsense, so I picked up his hat, handed it to him, and ordered him to clear out as fast as he could. I informed him, besides, that the nearest cut to the Charles River was through Exeter Street, just opposite my residence, and the sooner he drowned himself the better for the world and my peace of mind. This appeared to bring him to his senses, and he then begged me to help him in any way I thought best. I began by telling him that I had practiced medicine for twenty-five years and I had yet to give my first dose of any opiate. If he chose to act like a rational being and give his symptoms, I would soon find means to relieve him, and that without Morphine or kindred remedies, and ultimately cure him. The idiot had, it appears, put his penis and testicles in an ice-pack to relieve chordee, with the result that he soon had a constant desire to urinate and defecate, both of which operations gave him agonizing torture. He could not urinate unless by bending over and holding his legs wide apart. Chimaphila-umb. 30th potency relieved him in a comparatively short time, but he suffered a good deal still with a difficulty of urinating and passing stool, and with a feeling as if a large body were in the rectum, making sitting painful. On the 9th of July he was well enough to leave the city and fill a professional engagement. On the 23d of the same month I prescribed Nitric Acid$^{200}$ for him by mail, but as I have unfortunately mislaid his letter, I am unable to give the symptoms which led me to give him that remedy. On the 4th of September he came to the city, and my notes of that day read: "Patient looks much improved in physique. The discharge still profuse, thick and of light color. Some heat in urethra, during urination; no symptoms of prostatitis, but induration (stricture) of urethra still prominent." I gave him Natrum-muriat.$^{200}$, and for this reason: The induration must be due to the strong nitrate of silver injections he had used in his former attack, years ago. On the 8th of October my entry in note-book was: "Discharge of same character as on the 4th of September, but not so profuse; no pain during urination. The induration over location of stricture seems smaller. The stream of urine is increasing in size almost daily." Blanks were given the patient. My next report was November 2d: "Discharge continues, thick and tenacious, occasionally of a greenish hue; thickening of urethra still diminishing. Quite recently many of the old mental symptoms have returned. *Weak and languid.*" I now administered Hydrastis-canaden.$^{200}$, the debility and languor being the marked indication. The last notes I have of this case read: "February 2d, no discharge for weeks. No trace whatever of induration, although he believes he can detect its old location when he has an erection. The stream of urine is now normal." I saw this patient about ten days ago, when he told me he felt perfectly well, excepting when he ate too freely.

The third and last case I intend submitting to you offers but one important feature, and that is, in spite of the use of liquor, tobacco, and indulgences in coition, the patient was nevertheless cured in a short time.

The easy-going mortal who became my patient on November 21st, 1893, complained of frequent and painful urination; the pains were of a burning character, and the meatus was swollen and sensitive; the discharge, a scanty, glairy mucus. I gave him, as you have already anticipated, Cannabis-sat.$^{200}$. On the 27th his symptoms were: discharge, thick, greenish-yellow, and profuse, much increased at night. Sharp pains in glans penis, worse immediately after urinating; marked chordee. He received Mercurius-solub.$^{200}$. December 6th, he reported that for three days he had been free from pain and the discharge almost all gone; but after sleeping with a woman the night previous, he was not so well, some of the urinary irritation had returned and the discharge was again greenish and, in addition, slightly bloody at times. I repeated Mercurius-solub., but in the 3 M potency. Ten days later he reported himself as completely cured—drinking, smoking, gadding about, and "toying with Venus," notwithstanding.

I fear I have taken up a good deal of your valuable time and probably taxed your patience to the utmost; but ere I conclude, let me say that while I have stated that all the cases of gonorrhœa I ever had, with the exception of the few mentioned, have always yielded satisfactorily to the homœopathic remedy, let me assure you that I do not pretend that I have found it easy to prescribe. In fact, it has often been the very opposite, as I have good reason to remember. I have frequently devoted more than one hour's time to the study of differentiating between two or more remedies. But what I wish to assert more particularly, and I do it without any reserve and in the greatest honesty: Every case, no matter how severe the pain or grave the complications, can be reached by the simillimum. It is possible I may meet, ere long, with cases that will prove intractable to my endeavors to cure, but if I do, I shall continue firm in the belief that the trouble will not be owing to any fault of our infallible law of cure, but to its ignorant practitioner.

